# Prognostic Significance of the Systemic Immune-Inflammation Index (SII) in Patients With Small Cell Lung Cancer: A Meta-Analysis

**DOI:** 10.3389/fonc.2022.814727

**Published:** 2022-02-04

**Authors:** Yuting Zhou, Menglu Dai, Zongxin Zhang

**Affiliations:** Clinical Laboratory, Huzhou Central Hospital, Affiliated Central Hospital of Huzhou University, Huzhou, China

**Keywords:** systemic immune-inflammation index, meta-analysis, prognosis, small cell lung cancer, biomarker

## Abstract

**Background:**

Previous studies have investigated the prognostic value of the systemic immune-inflammation index (SII) in small cell lung cancer (SCLC). However, the results have been inconsistent. The study aimed to investigate the prognostic and clinicopathological significance of SII in SCLC through a meta-analysis.

**Methods:**

The PubMed, Web of Science, Embase, Cochrane Library, and China National Knowledge Infrastructure databases were thoroughly searched. The pooled hazard ratios (HRs) with 95% confidence intervals (CIs) were calculated to evaluate the prognostic value of the SII for survival outcomes. The combined odds ratios (ORs) and 95% CIs were used to evaluate the correlation between SII and clinicopathological features.

**Results:**

Eight studies comprising 2,267 patients were included in the meta-analysis. Pooled analyses indicated that a high SII was significantly associated with worse overall survival (OS) (HR=1.52, 95% CI=1.15–2.00, p=0.003) but not progression-free survival (HR=1.38, 95% CI=0.81–2.35, p=0.238) in patients with SCLC. Moreover, a high SII was associated with extensive-stage SCLC (OR=2.43, 95% CI=1.86–3.17, p<0.001). However, there was a non-significant correlation between SII and age, sex, smoking history, Karnofsky Performance Status score, or initial therapeutic response.

**Conclusion:**

Our meta-analysis demonstrated that a high SII could be an efficient prognostic indicator of OS in SCLC. We recommend adopting SII to predict OS in patients with SCLC, and SII in combination with other parameters or biomarkers may aid in addressing the clinical strategy and choosing the best treatment for an individual patient.

## Introduction

Lung cancer is the leading cause of cancer-related deaths globally, with 2,093,876 new cases diagnosed and 1,761,007 deaths annually (1). Small cell lung cancer (SCLC) is an aggressive malignancy that accounts for 15% of all lung cancer cases and causes more than 200,000 deaths per year (2). SCLC is a highly metastatic tumor strongly associated with smoking (3). SCLC is usually classified as a limited and extensive-stage disease (LS-SCLC and ES-SCLC). Approximately 70% of patients with SCLC have ES-SCLC at diagnosis (4). The prognosis of SCLC is poor. The 1-year and 2-year overall survival (OS) rates in LS-SCLC were 58% and 21%, respectively, and they were 29.4% and 7%, respectively, for ES-SCLC (5).

Combination chemoradiotherapy followed by maintenance immunotherapy is the new standard of care for the upfront management of metastatic SCLC (6). A recent report of IMpower133 (ClinicalTrials.gov identifier: NCT02763579) showed that adding atezolizumab to carboplatin plus etoposide as the first-line treatment for ES-SCLC continued to demonstrate improved OS and a tolerable safety profile in the updated analysis, confirming the regimen as a new standard of care (7). The standard treatment for LS-SCLS is combined modality treatment, including surgery, radiation, and systemic therapy (6). Surgical resection is recommended for eligible patients with early-stage (I–IIA, T1–2N0) disease who have undergone pathologic mediastinal staging to exclude nodal involvement (6). For patients with stage IIB to IIIC (T1–T4N0–N3M0) disease, the standard of care is management with concurrent platinum-based chemotherapy and radiotherapy (6). Despite these advances in the past several decades, the survival of SCLC has not substantially improved. Therefore, it is crucial to identify reliable and novel prognostic markers for SCLC.

Recent studies have shown immunological biomarkers, such as neutrophil-to-lymphocyte ratio, platelet-to-lymphocyte ratio, C-reactive protein/albumin ratio, and systemic immune-inflammation index (SII), have prognostic roles in a series of malignant tumors (8, 9). SII is calculated based on peripheral neutrophil, platelet, and lymphocyte counts using the following formula: platelet count × neutrophil count/lymphocyte count (10). SII has been reported as a significant prognostic biomarker for various cancers, including hepatocellular carcinoma (HCC) (11), gastric cancer (12), pancreatic cancer (13), endometrial cancer (14), non-small cell lung cancer (15–17), and bladder cancer (18). Recent studies have also investigated the prognostic value of SII in patients with SCLC; however, the results remain inconsistent (19–26). For example, some studies showed that a high SII was associated with worse survival in SCLC (21, 23), whereas others have not identified the prognostic value of SII (20, 26). Therefore, we performed a systematic and comprehensive meta-analysis to identify the prognostic and clinicopathological significance of SII in SCLC.

## Materials and Methods

### Literature Search

The current meta-analysis was conducted according to the Preferred Reporting Items for Systematic Reviews and Meta-Analyses statement (27). The PubMed, Web of Science, Embase, Cochrane Library, and China National Knowledge Infrastructure databases were thoroughly searched. The following search items and texts were used: (“systemic immune-inflammation index” OR “SII”) AND (“small cell lung cancer” OR “SCLC”). The last search was updated on October 16, 2021. There were no limitations to the publication language. Additionally, the reference lists of pertinent articles were manually searched for potentially eligible studies.

### Inclusion and Exclusion Criteria

The inclusion criteria were as follows (1): patients pathologically diagnosed with SCLC; (2) the articles investigated the prognostic role of SII for survival outcomes, including OS or progression-free survival (PFS); (3) there was no limitation to the treatment methods, if only the treatment for patients was applied according to the standard treatment guidelines, including surgery, chemotherapy alone, immunotherapy alone, and concurrent chemoradiotherapy; (4) platelet counts, neutrophil counts, and lymphocyte counts were measured using serum-based methods before treatment; (5) a cut-off value of SII was identified; (6) hazard ratios (HRs) with 95% confidence intervals (CIs) for survival outcomes were reported in text or can be extracted from Kaplan–Meier curves; and (7) published in English or Chinese. The exclusion criteria were: (1) meeting abstracts, letters, case reports, reviews, or comments; (2) studies with insufficient data for analysis; (3) animal studies; and (4) studies that included overlapping patients. The primary endpoint was OS, defined as the period from diagnosis until death from any cause and the last follow-up period for living patients. The secondary endpoint was PFS, which was determined as the time interval from diagnosis to progression or death.

### Data Extraction and Quality Assessment

Two investigators (Y.Z. and M.D.) independently reviewed all studies, and all discrepancies were resolved by discussion with a third investigator (Z.Z.) until consensus was reached. The following data were extracted from each qualified study: name of the first author, year of publication, country, study period, study design, age, Veterans Administration Lung Study Group stage, treatment, follow-up, a cut-off value of SII, determination method of cut-off value, survival outcomes, survival analysis (multivariate or univariate), HRs, and 95% CIs. If both multivariate and univariate analyses were performed, the HRs and 95% CIs of the multivariate analysis were adopted. The methodological quality of the included studies was evaluated using the Newcastle–Ottawa Scale (NOS) for cohort studies (http://www.ohri.ca/programs/clinical_epidemiology/oxford.asp) by two independent authors (Y.Z. and M.D.). The NOS evaluates the quality of studies in three aspects: selection (0-4 points), comparability (0-2 points), and outcome (0-3 points). The NOS scores range from 0-9, and studies with NOS scores > 6 were considered high quality.

### Statistical Analysis

The pooled HRs and 95% CIs were calculated to evaluate the prognostic value of the SII for survival outcomes in patients with SCLC. The heterogeneity among studies was assessed using the Cochrane Q test and *I* ^2^ statistic. A fixed-effects model was used in the absence of significant heterogeneity (*I* ^2^<50% or P for heterogeneity >0.10); otherwise, a random-effects model was utilized. The combined odds ratios (ORs) and 95% CIs were used to evaluate the correlation between SII and clinicopathological features in SCLC. Subgroup analysis was conducted to detect the source of heterogeneity and for further investigation. Publication bias was evaluated visually using Begg’s funnel plot and Egger’s test. All statistical analyses were conducted using Stata 12.0 software (Stata Corp LP, Texas, USA). All statistical tests were two-sided, and statistical significance was defined as p<0.05.

### Ethics

The requirement for ethical approval and informed consent was waived because all analyses in this study were based on previously published reports.

## Results

### Search Results

**Figure 1:**
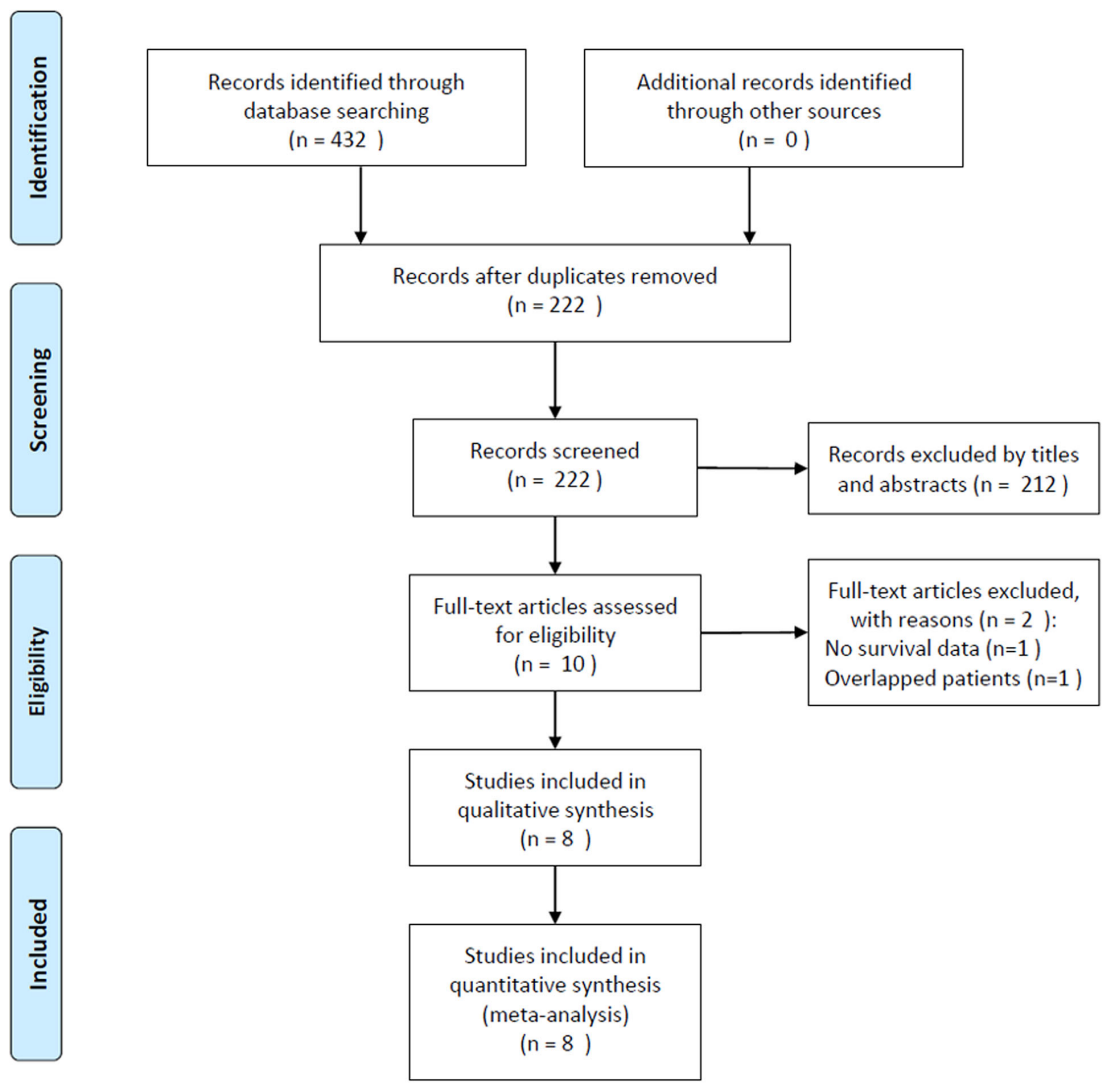
Flow diagram of included studies for this meta-analysis.

The initial literature search identified 432 studies, and 222 records remained after excluding duplicate studies. After screening the titles and abstracts, 212 studies were removed, and 10 studies were reviewed in full text. Subsequently, two studies were eliminated for the following reasons: one study did not provide survival data and one recruited overlapping patient. Finally, eight studies comprising 2,267 patients (19-26) were included in this meta-analysis. The detailed study selection process is shown in **Figure 1**.

### Baseline Characteristics of Included Studies

The characteristics of the included studies are summarized in **Table 1**. The included studies were published between 2015 and 2021. Seven studies were conducted in China (19–25) and one study was conducted in Turkey (26). The sample size ranged from 41 to 919 (median, 157). Seven studies were published in English (19, 20, 22–26), and one was published in Chinese (21). Six studies were retrospective (19, 21–24, 26), and two were prospective trials (20, 25). Six studies (19–23, 26) investigated the prognostic role of SII for OS, and five studies (21, 23–26) explored the association between SII and PFS. Seven studies (19, 21–26) recruited both LS-SCLS and ES-SCLC, and one study (20) only enrolled patients with ES-SCLC. The cut-off values of SII ranged from 479 to 1600, with a median value of 673. Six studies (20–23, 25, 26) used receiver operating characteristic curve analysis to determine the cut-off value, and two studies (19, 24) referred to the literature. HRs and 95% CIs were extracted from multivariate analysis in five studies (19, 20, 22, 23, 26) and univariate analysis in three studies (21, 24, 25). The NOS scores of the included studies ranged from 7 to 9, indicating that all included studies were of high quality. The details of the NOS scores are summarized in **Supplementary File 1**.

**Table 1:** Main characteristics of the included studies in this analysis.

Study	Year	Country	Sample size	Study period	Study design	Sex (F/M)	Age, years median(range)	VALG stage (LS/ES)	Treatment	Follow-up (month)Median(range)	Cut-off value of SII	Cut-off determination	Survival outcomes	Survival analysis	NOS score
Hong	2015	China	919	2000-2012	Retrospective	284/635	56(16-84)	552/367	CRT	To Dec 2014	1600	Literature	OS	Multivariate	7
Qi	2021	China	53	2017-2018	Prospective	19/34	65	0/53	Chemotherapy+ Targeted therapy	17.1	533	ROC analysis	OS	Multivariate	9
Teng	2021	China	98	2013-2018	Retrospective	36/62	60	50/48	CRT	To Jun 2020	571.5	ROC analysis	OS, PFS	Univariate	8
Wang	2020	China	653	2008-2009	Retrospective	231/422	56 (23-75)	384/269	CRT	NR	748.5	ROC analysis	OS	Multivariate	7
Wang	2019	China	228	2009-2015	Retrospective	69/159	58 (39-71)	114/114	CRT	46	479	ROC analysis	OS, PFS	Multivariate	8
Xiong	2021	China	41	2015-2018	Retrospective	5/36	61	7/34	Immunotherapy	To Aug 2019	730	Literature	PFS	Univariate	8
Yao	2021	China	59	2018-2020	Prospective	14/45	63 (45-78)	35/24	Chemotherapy	9.1 (1.5-24.2)	720	ROC analysis	PFS	Univariate	7
Yilmaz	2020	Turkey	216	2010-2019	Retrospective	32/184	61 (36-83)	59/157	CRT	10 (1-74)	626	ROC analysis	OS, PFS	Multivariate	8

VALG, Veterans Administration Lung Study Group; F, female; M, male; LS, limited stage; ES, extensive stage; CRT, chemoradiotherapy; OS, overall survival; PFS, progression-free survival; NOS, Newcastle-Ottawa Scale; ROC, receiver operating characteristic curve; SII, systemic immune-inflammation index.

### Impact of SII on Overall Survival in SCLC

**Figure 2:**
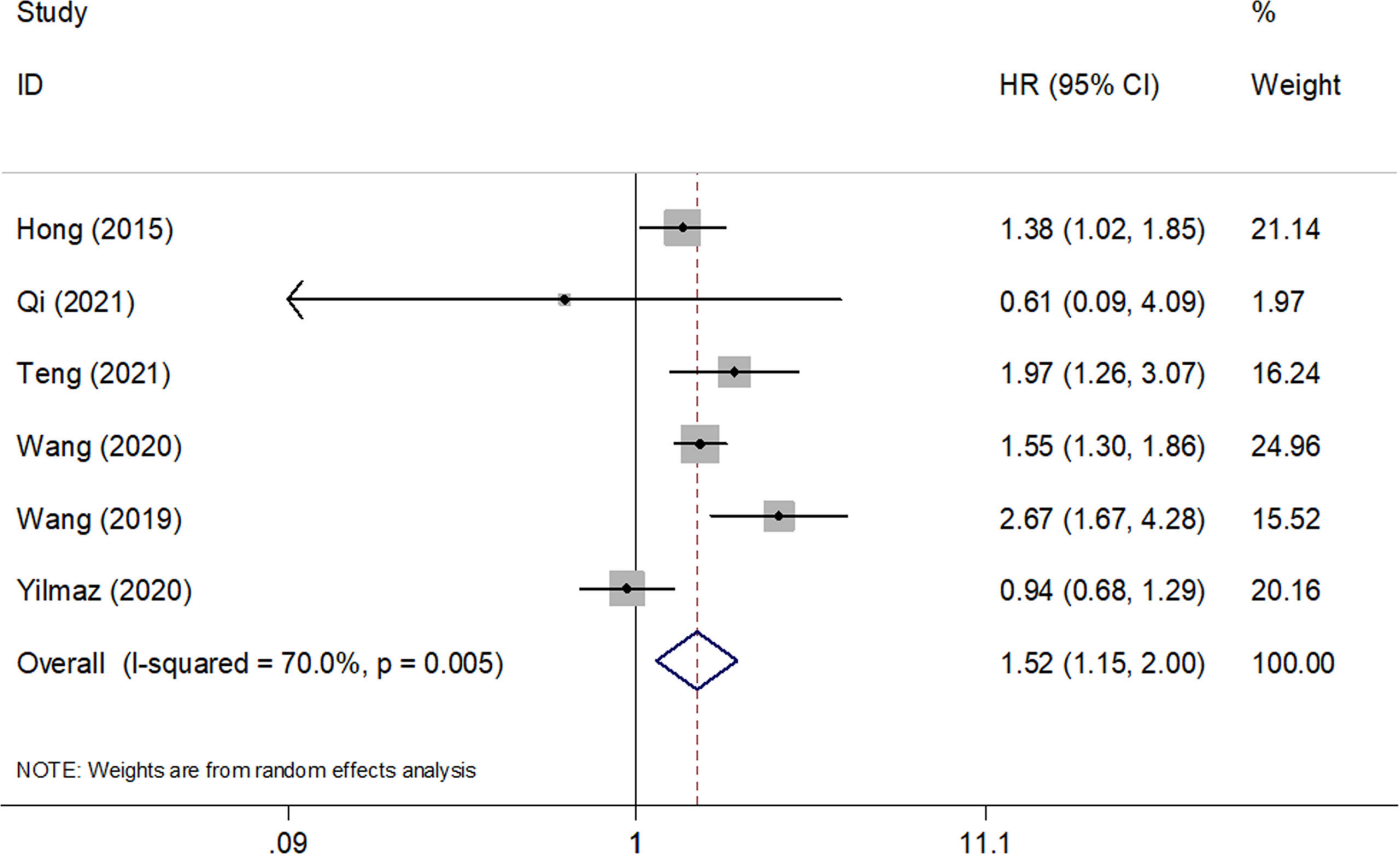
Forest plots of pooled HRs and associated 95% CIs of the effect of high versus low SII for overall survival in patients with SCLC.

A total of six studies with 2,167 patients (19–23, 26) reported the prognostic value of SII for OS in SCLC. A random-effects model was applied because of significant heterogeneity (*I* ^2 =^ 70%, Ph=0.005). As shown in **Figure 2** and **Table 2**, the pooled HR and 95% CI were HR= 1.52, 95% CI= 1.15–2.00, p=0.003, indicating that a high SII was associated with poor OS. Subgroup analysis stratified by country, sample size, study design, cut-off value, cut-off determination, survival analysis, tumor stage, and treatment were performed. The results demonstrated that a high SII remained a prognostic factor for OS in Chinese patients, with a cut-off value of ≥700, and the prognostic role was not influenced by the cut-off determination method. In addition, as shown in **Table 2**, elevated SII was associated with poor OS in patients with LS + ES but not in patients with ES.

**Table 2:** Subgroup analysis of the prognostic value of SII for overall survival in patients with SCLC.

Variables	No. of studies	No. of patients	Effects model	HR (95%CI)	p	Heterogeneity	
						*I* ^2^(%)	Ph
Total	6	2,167	Random	1.52 (1.15-2.00)	0.003	70.0	0.005
Country							
China	5	1,951	Fixed	1.61 (1.41-1.85)	<0.001	46.5	0.113
Turkey	1	216	–	0.94 (0.68-1.29)	0.685	–	–
Sample size							
<200	2	151	Fixed	2.19 (1.59-3.01)	<0.001	24.2	0.267
≥200	4	2,016	Random	1.29 (0.97-1.72)	0.084	72.2	0.027
Study design							
Prospective	1	53	–	0.61 (0.09-4.11)	0.612	–	–
Retrospective	5	2,114	Random	1.55 (1.16-2.05)	0.003	74.7	0.003
Cut-off value of SII							
<700	4	595	Random	1.54 (0.83-2.85)	0.170	81.4	0.001
≥700	2	1,572	Fixed	1.50 (1.29-1.75)	<0.001	0	0.495
Cut-off determination							
ROC analysis	5	1,248	Random	1.56 (1.08-2.25)	0.018	75.5	0.003
Literature	1	919	–	1.38 (1.02-1.85)	0.034	–	–
Survival analysis							
Multivariate	5	2.069	Random	1.44 (1.05-1.98)	0.023	73.3	0.005
Univariate	1	98	–	1.97 (1.26-3.07)	0.003	–	–
Treatment							
CRT	5	1,886	Random	1.39 (1.07-1.81)	0.013	68.1	0.024
C+T/C/I	2	281	Random	1.73 (0.46-6.47)	0.418	53.9	0.141
Tumor stage							
LS+ES	5	2,114	Random	1.55 (1.16-2.05)	0.003	74.7	0.003
ES	1	53	–	0.61 (0.09-4.11)	0.612	–	–

SII, systemic immune-inflammation index; ROC, receiver operating characteristic curve; CRT, chemoradiotherapy; C+T/C/I, Chemotherapy + Targeted therapy/Chemotherapy/Immunotherapy.

### Impact of SII on Progression-Free Survival in SCLC

**Figure 3:**
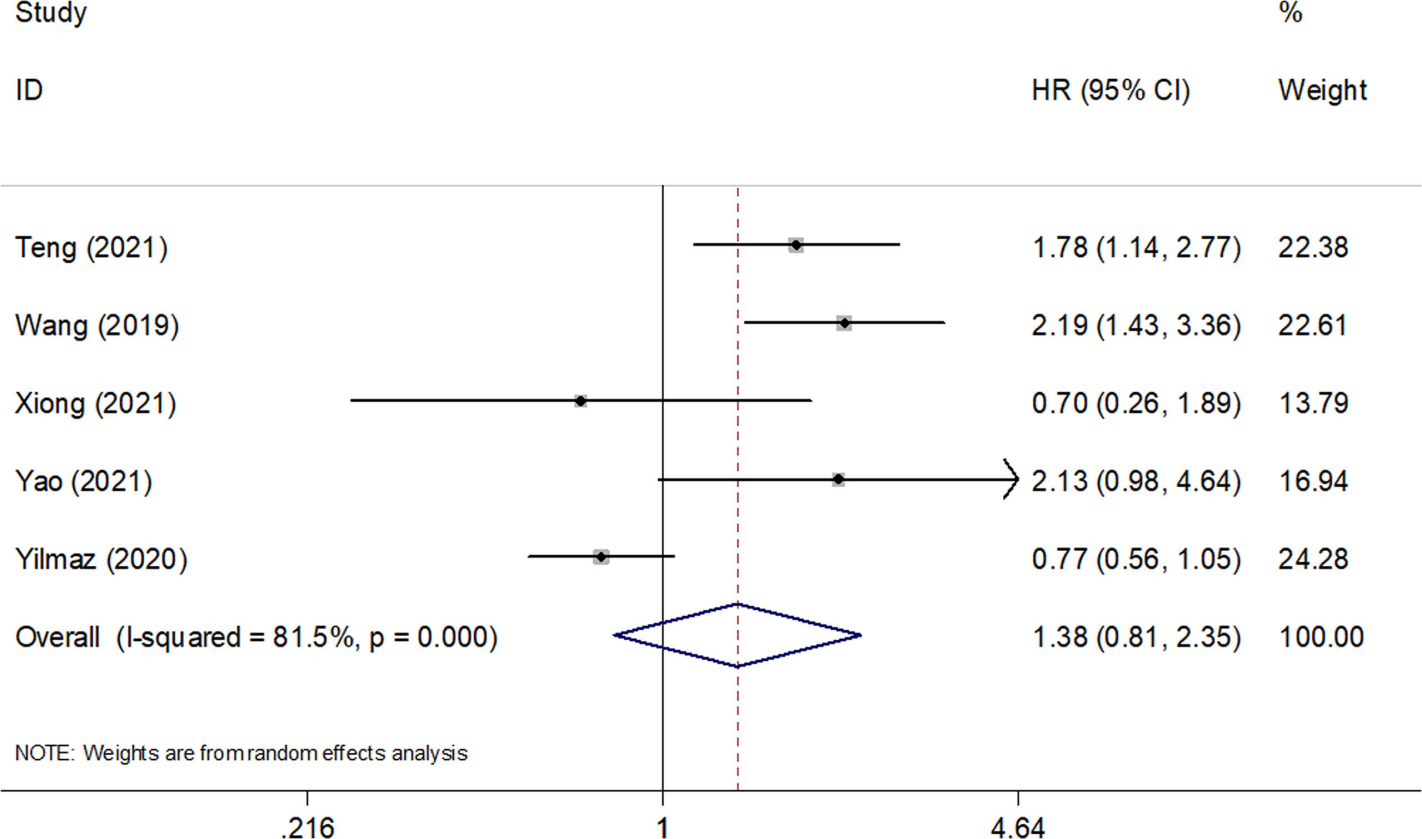
Forest plots of pooled HRs and associated 95% CIs of the effect of high versus low SII for progression-free survival in patients with SCLC.

Five studies consisting of 642 patients (21, 23–26) investigated the prognostic significance of the SII for PFS in SCLC. The combined HR and 95% CI were HR=1.38, 95% CI=0.81–2.35, p=0.238 (**Table 3** and **Figure 3**), which suggested that SII was not associated with PFS in patients with SCLC. The subgroup analysis suggested that elevated SII was a significant prognostic marker for poor PFS in Chinese patients with SCLC (HR=1.85, 95% CI=1.40–2.43, p<0.001; **Table 3**).

**Table 3:** Subgroup analysis of the prognostic value of SII for progression-free survival in patients with SCLC.

Variables	No. of studies	No. of patients	Effects model	HR (95%CI)	p	Heterogeneity	
						*I* ^2^(%)	Ph
Total	5	642	Random	1.38 (0.81-2.35)	0.238	81.5	<0.001
Country							
China	4	426	Fixed	1.85 (1.40-2.43)	<0.001	32.4	0.218
Turkey	1	216	–	0.77 (0.56-1.05)	0.093	–	–
Sample size							
<200	3	198	Fixed	1.64 (1.14-2.34)	0.007	41.2	0.182
≥200	2	444	Random	1.28 (0.46-3.58)	0.637	93.4	<0.001
Study design							
Prospective	1	59	–	2.13 (0.98-4.64)	0.056	–	–
Retrospective	4	583	Random	1.26 (0.69-2.30)	0.456	84.8	<0.001
Cut-off value of SII							
<700	3	542	Random	1.42 (0.71-2.82)	0.318	89.2	<0.001
≥700	2	100	Random	1.28 (0.43-3.80)	0.659	66.8	0.083
Cut-off determination							
ROC analysis	4	601	Random	1.54 (0.85-2.77)	0.151	85.2	<0.001
Literature	1	41	–	0.70 (0.26-1.89)	0.481	–	–
Survival analysis							
Multivariate	2	444	Random	1.28 (0.46-3.58)	0.637	93.4	<0.001
Univariate	3	198	Fixed	1.64 (1.14-2.34)	0.007	41.2	0.182
Treatment							
CRT	3	542	Random	1.42 (0.71-2.82)	0.318	89.2	<0.001
C+T/C/I	2	100	Random	1.28 (0.43-3.80)	0.659	66.8	0.083

SII, systemic immune-inflammation index; ROC, receiver operating characteristic curve; CRT, chemoradiotherapy; C+T/C/I, Chemotherapy + Targeted therapy/Chemotherapy/Immunotherapy.

### The Correlation Between SII and Clinicopathological Factors in SCLC

**Figure 4:**
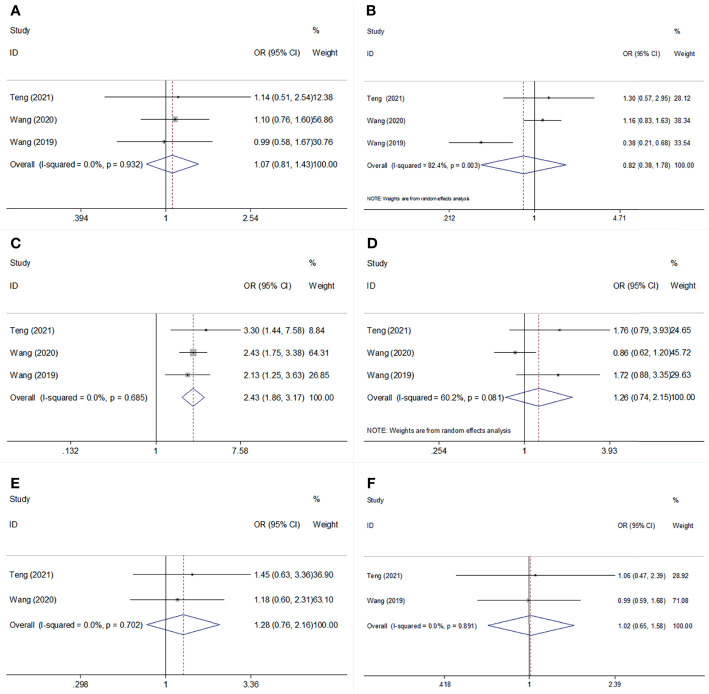
Forest plot for the association of SII with age, sex, stage, smoking status, KPS score, and Initial therapeutic response in SCLC. **(A)** age; **(B)** sex: **(C)** stage: **(D)** smoking status; **(E)** KPS score; **(F)** Initial therapeutic response.

We investigated the association between SII and clinicopathological features, including age (≥60 vs <60 years), sex (male vs female), stage (ES vs LS), smoking history (yes vs no), Karnofsky Performance Status (KPS) score (< 80 vs ≥80), and initial therapeutic response (stable disease + progressive disease vs complete response + partial response) in SCLC. As shown in **Figure 4** and **Table 4**, a high SII was associated with ES-SCLC (OR=2.43, 95% CI=1.86–3.17, p<0.001). However, there was a non-significant correlation between SII and age, sex, smoking history, KPS score, or initial therapeutic response (**Figure 4**; **Table 4**).

**Table 4:** The correlation between SII and clinicopathological features in patients with SCLC.

Clinicopathological factors	No. of studies	No. of patients	Effects model	OR (95%CI)	p	Heterogeneity	
						*I* ^2^(%)	Ph
Age (years) (≥60 vs <60)	3	979	Fixed	1.07 (0.81-1.43)	0.633	0	0.932
Sex (male vs female)	3	979	Random	0.82 (0.38-1.78)	0.619	82.4	0.003
Stage (ES vs LS)	3	979	Fixed	2.43 (1.86-3.17)	<0.001	0	0.685
Smoking history (yes vs no)	3	979	Random	1.26 (0.74-2.15)	0.397	60.2	0.081
KPS score (<80 vs ≥80)	2	751	Fixed	1.28 (0.76-2.16)	0.355	0	0.702
Initial therapeutic response (SD + PD vs CR + PR)	2	326	Fixed	1.02 (0.65-1.58)	0.947	0	0.891

SII, systemic immune-inflammation index; LS, limited stage; ES, extensive stage; KPS, Karnofsky Performance Status; SD, Stable disease; PD, Progressive disease; CR, Complete response; PR, Partial response.

### Publication Bias

**Figure 5:**
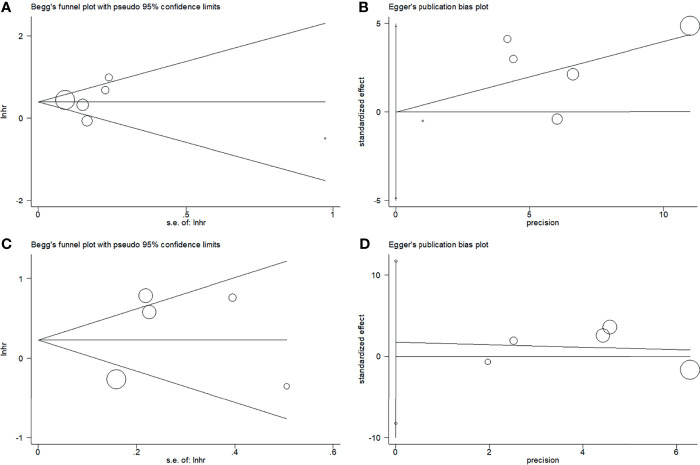
Plots for publication bias test. **(A)** Begg’s funnel plot for OS, p=0.851; **(B)** Egger’s publication bias plot for OS, p=0.223; **(C)** Begg’s funnel plot for PFS, p=0.806; **(D)** Egger’s publication bias plot for PFS, p=0.617.

Begg’s funnel plots and Egger’s test were used to estimate the potential publication bias. The results showed that there was no significant publication bias for OS (Begg’s test: p=0.851; Egger’s test: p=0.223) or PFS (Begg’s test: p=0.806; Egger’s test: p=0.617) (**Figure 5**).

## Discussion

SCLC is a highly malignant carcinoma with a poor prognosis because of the elusive pathophysiology of the disease (28). Previous studies have investigated the prognostic effect of SII in SCLC; however, the conclusions are not consistent. In the present meta-analysis, data from eight studies with 2,267 patients were combined, and the results showed that an elevated SII was associated with worse OS, but not PFS. Furthermore, subgroup analysis indicated that a high SII was a significant prognostic factor for poor OS and PFS in Chinese patients with SCLC. Furthermore, a high SII was significantly correlated with ES-SCLC, suggesting that SII could indicate metastasis in SCLC. Our meta-analysis demonstrated that SII could be applied as an effective prognostic index for poor OS in SCLC, especially in Chinese patients. To our knowledge, this is the first meta-analysis to investigate the prognostic and clinicopathological significance of SII in patients with SCLC.

SII was first identified as a useful prognostic indicator in patients with HCC in 2014 (11). SII is calculated as neutrophil × platelet/lymphocyte and is cost-effective and easily accessible. A high SII could be attributed to high neutrophil counts, high platelet counts, or low lymphocyte counts. The exact mechanisms of the prognostic value of SII in SCLC have not been fully elucidated and can be explained in the following aspects. First, neutrophils secrete cytokines and chemokines, including vascular epidermal growth factor (VEGF), to enhance tumor angiogenesis and facilitate distant metastasis (29). Second, previous studies have shown that platelets play a crucial role in tumor activity. Platelets can mediate the survival and growth of tumor cells by secreting a various cytokines, such as VEGF, transforming growth factor-β, and platelet-derived growth factor (30). In addition, platelet-associated chemokines can modulate immune responses in the tumor environment and tumor angiogenesis (31). Third, lymphocytes are critically involved in cancer immune surveillance to prevent tumor development (32). Tumor-infiltrating lymphocytes are important immune cells in the tumor microenvironment and are responsible for antitumor immune responses (33). Lymphocytes play a vital role in immune defense against tumor cells, including inhibition of tumor cell proliferation and metastasis (34). Therefore, a high SII could be applied as a reliable biomarker of tumor progression and poor prognosis.

In addition, a high SII might be a consequence of a high tumor burden/metastatic/diffuse disease, which is the cause of tumor progression. For example, high SII, resulting from neutrophilia, lymphopenia, and thrombocytosis, may also be a useful prognostic indicator for postoperative survival outcomes (35) and for estimating response rates in cancer patients treated with chemotherapy (36) and immunotherapy (37). The SII is also a powerful tool for predicting outcomes in diffuse large B-cell lymphoma (10).

Several studies have shown the prognostic value of SII in various cancers through meta-analysis (38–40). For example, Qiu et al. showed that a high pretreatment SII predicted poor OS but not poor disease-free survival (DFS) in patients with gastric cancer, based on a meta-analysis of eight studies (38). Shui et al. reported that elevated SII was associated with poor OS, recurrence-free survival (RFS)/PFS/DFS, and cancer-specific survival in patients with pancreatic cancer in a meta-analysis including 2,365 subjects (39). In addition, Zhang et al. demonstrated that breast cancer patients with a high SII had worse OS, poorer DFS/RFS, and inferior distant metastasis-free survival than patients with a low SII (41). A recent meta-analysis of 12 studies showed that an elevated SII index was significantly associated with poor OS, PFS, and CSS in patients with urinary system cancers (40). In the current meta-analysis, we identified a significant prognostic role of SII for OS but not for PFS. A possible reason is that the PFS is usually shorter than the OS in each study. Therefore, the difference in prognosis for PFS could not be significant in a relatively short duration.

There are some limitations to this meta-analysis need to be noted. First, the patients included in the meta-analysis were from Asia, mainly China. Therefore, our results apply to Asian patients. Second, the sample size was relatively small. Although eight studies were included, the total sample size was 2,267. Only six studies were included for OS analysis and five studies for PFS analysis. Third, most of the included studies were retrospective, and only two studies were prospective, which may have led to selection bias. Therefore, large-scale prospective trials including diverse populations are needed to validate the results of our meta-analysis.

In summary, our meta-analysis demonstrated that an elevated SII was associated with poor OS in patients with SCLC. Moreover, a high SII was predictive of ES-SCLC. We recommend adopting SII to predict OS in patients with SCLC, and SII in combination with other parameters or biomarkers may aid in addressing the clinical strategy and choosing the best treatment for each patient. Due to the limitations mentioned above, further large-scale prospective trials are needed to validate our findings.

## Data Availability Statement

The original contributions presented in the study are included in the article/**Supplementary Material**. Further inquiries can be directed to the corresponding authors.

## Author Contributions

YZ and MD designed and supervised the study. ZZ drafted the manuscript, carried out the literature search, and extracted the data from the eligible studies. YZ and ZZ contributed to the quality control of study inclusion and discussion. All authors contributed to data analysis, drafting and revising the article, gave final approval of the version to be published, and agree to be accountable for all aspects of the work. All authors read and approved the final manuscript.

## Conflict of Interest

The authors declare that the research was conducted in the absence of any commercial or financial relationships that could be construed as a potential conflict of interest.

## Publisher’s Note

All claims expressed in this article are solely those of the authors and do not necessarily represent those of their affiliated organizations, or those of the publisher, the editors and the reviewers. Any product that may be evaluated in this article, or claim that may be made by its manufacturer, is not guaranteed or endorsed by the publisher.
