# Supplementary material for: Prognostic Significance of the Systemic Immune-Inflammation Index (SII) in Patients With Small Cell Lung Cancer: A Meta-Analysis
Source: Front Oncol. 2022 Feb 4;12:814727. doi: 10.3389/fonc.2022.814727 (PMC8854201; doi:10.3389/fonc.2022.814727)
Supplement: Supplementary file 1 [file Table_1.docx]

Supplementary Table 1 The quality assessment scores by NOS of included studies in our meta-analysis.

| Study | Year | Selection (0-4 points) | | | | Comparability  (0-2 points) | Outcome  (0-3 points) | | | Total score |
| --- | --- | --- | --- | --- | --- | --- | --- | --- | --- | --- |
|  |  | Representativeness of the exposed cohort | Selection of the non exposed cohort | Ascertainment of exposure | Demonstration that outcome of interest was not present at start of study | Comparability of cohorts on the basis of the design or analysis | Assessment of outcome | Was follow-up long enough for outcomes to occur | Adequacy of follow up of cohorts |  |
| Hong | 2015 | ★ | ★ | ☆ | ★ | ★★ | ★ | ★ | ☆ | 7 |
| Qi | 2021 | ★ | ★ | ★ | ★ | ★★ | ★ | ★ | ★ | 9 |
| Teng | 2021 | ★ | ★ | ★ | ★ | ★★ | ★ | ☆ | ★ | 8 |
| Wang | 2020 | ★ | ★ | ★ | ★ | ★★ | ★ | ☆ | ☆ | 7 |
| Wang | 2019 | ★ | ★ | ★ | ★ | ★☆ | ★ | ★ | ★ | 8 |
| Xiong | 2021 | ★ | ★ | ★ | ★ | ★★ | ★ | ★ | ☆ | 8 |
| Yao | 2021 | ★ | ★ | ★ | ★ | ★☆ | ★ | ☆ | ★ | 7 |
| Yilmaz | 2020 | ★ | ★ | ★ | ★ | ★☆ | ★ | ★ | ★ | 8 |

NOS: Newcastle-Ottawa Scale; a ★ represents 1 point; a ☆ represents 0 point.
